# Line-Field Confocal Optical Coherence Tomography for the Diagnosis of Skin Tumors: A Systematic Review and Meta-Analysis

**DOI:** 10.3390/diagnostics14141522

**Published:** 2024-07-15

**Authors:** Shazli Razi, Yen-Hong Kuo, Gaurav Pathak, Priya Agarwal, Arianna Horgan, Prachi Parikh, Farah Deshmukh, Babar K. Rao

**Affiliations:** 1 Department of Internal Medicine, Hackensack Meridian Ocean University Medical Center, Brick, NJ 08724, USA; 2 Department of Internal Medicine, Jersey Shore University Medical Center, Neptune, NJ 07753, USA; 3 Office of Research Administration, Hackensack Meridian Health Research Institute, Nutley, NJ 07110, USA; 4 Department of Medical Sciences, Hackensack Meridian School of Medicine, Nutley, NJ 07110, USA; 5 Center for Dermatology, Robert Wood Johnson Medical School, Rutgers University, New Brunswick, NJ 08901, USA; 6 Department of Dermatology, Rao Dermatology, Atlantic Highlands, NJ 07716, USA; 7 Department of Dermatology, Weill Cornell Medicine, New York, NY 10021, USA

**Keywords:** line-field confocal optical coherence tomography, basal cell carcinoma, squamous cell carcinoma, melanoma

## Abstract

A line-field confocal optical coherence tomography (LC-OCT) combines confocal microscopy and optical coherence tomography into a single, rapid, easy-to-use device. This meta-analysis was performed to determine the reliability of LC-OCT for diagnosing malignant skin tumors. PubMed, EMBASE, Web of Science databases, and the Cochrane Library were searched for research studies in the English language from inception till December 2023. To assess quality and the risk of bias, the Quality Assessment of Diagnostic Accuracy Studies-2 (QUADAS-2) was used. The sensitivity and specificity of each study were calculated. The bivariate summary sensitivity and specificity were calculated using the linear mixed model. Five studies with 904 reported per lesion analyses in our study; the specificity and sensitivity ranged from 67% to 97% and 72% to 92%, respectively. The pooled specificity and sensitivity were 91% (95% CI: 76–97%) and 86.9% (95% CI: 81.8–90.8%), respectively. The summary sensitivity and specificity from the bivariate approach are 86.9% (95% CI: 81.8–90.8%) and 91.1% (95% CI: 76.7–97.0%), respectively. The area under the curve is 0.914. LC-OCT shows great sensitivity and specificity in diagnosing malignant skin tumors. However, due to the limited number of studies included in our meta-analysis, it is premature to elucidate the true potential of LC-OCT.

## 1. Introduction

**Figure 1:**
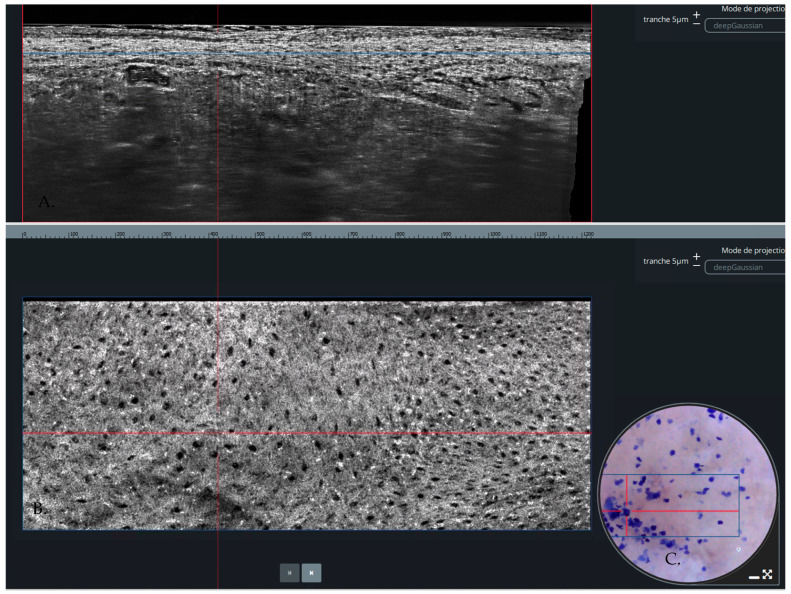
(**A**) Vertical view of LC-OCT (**B**) Horizontal or Enface view of LC-OCT. (**C**) Dermoscopic view of the lesion.

Line-field confocal optical coherence tomography (LC-OCT) is a non-invasive, real-time, high-resolution visualization technique of the epidermis and upper dermis. A relatively recent innovation, LC-OCT generates a 3-dimensional structural representation from a series of vertical and horizontal images [1]. With a resolution of up to ~1 µm, LC-OCT surpasses the achievable resolution of conventional OCT (5 to 10 µm) [2,3]. Further, LC-OCT has a penetration depth of ~500 µm as compared to 200 µm with reflectance confocal microscopy (RCM) [2,4]. Currently, LC-OCT is used less commonly than dermoscopy, RCM, histopathology, and conventional OCT as a screening or diagnostic tool. Despite relatively low utilization by practitioners, LC-OCT has demonstrated proficiency in the diagnosis of various skin conditions (Figure 1, example of a lesion visualized under LC-COT). In particular, LC-OCT is becoming an increasingly popular tool in the recognition of basal cell carcinoma (BCC) thanks in part to its ability to distinguish between histopathological subcategories of BCC [2].

Despite the availability of empirically validated diagnostic and screening tools, LC-OCT offers multiple unique advantages. Notably, LC-OCT is completely non-invasive and can be completed in a matter of minutes. This is in stark contrast with skin biopsy, which is currently the most common technique employed in the diagnosis of suspicious skin lesions. Although regarded as safe and effective, skin biopsies are invasive, time-consuming, and usually yield benign results [1]. Furthermore, skin biopsies account for 30% of the visit cost for a total body check [5]. LC-OCT, therefore, has the potential to be a more economical alternative to skin biopsy. Given that incidence rates of skin cancer are climbing, a cost-effective screening/diagnostic tool is of pivotal importance. Currently, 20% of Americans will be diagnosed with skin cancer in their lifetime, and the prevalence of melanoma and non-melanoma skin cancer (NMSC) is increasing [6,7]. In the past 30 years alone, the incidence of SCC in the U.S. has increased by 3–10% every year, and the incidence of BCC has increased by between 20 and 80% [7]. Between 2012–2015 and 2016–2018, the annual cost of skin cancer treatment rose from USD 8 billion to 8.9 billion [8]. Accordingly, the early detection of skin cancers remains an invaluable asset in minimizing economic losses, morbidity, and mortality.

Evidence from previous studies has suggested that LCT-OCT can be an effective, non-invasive tool to differentiate between cancerous and benign skin lesions. Integrating LCT-OCT as an assessment for skin lesions has the potential to increase diagnostic accuracy and identify a less invasive tool for detecting skin cancer, reducing patient burden or risk of skin biopsies and allowing early skin cancer diagnoses. In order to better understand the role of LC-OCT, we conducted a meta-analysis to assess the accuracy of LC-OCT as a clinical diagnostic aid for malignant skin tumors non-invasively.

## 2. Materials and Methods

### 2.1. Literature Search

We searched PubMed, EMBASE, Web of Science databases, and the Cochrane Library for research studies in the English language from inception till December 2023. The following keywords were searched, separately and in combination: ‘basal cell carcinoma’, ‘BCC’, ‘squamous cell carcinoma’, ‘SCC’, ‘skin cancer’, ‘tumor’, ‘nevus’, ‘melanoma’, ‘Line-field’, ‘Line field confocal optical coherence tomography’, ‘LC-OCT’ and ‘LCOCT’. Each article was initially screened by title and abstract. Articles were provisionally included if any of the utilized key terms were found in their title or abstract. Following the initial screening, pertinent articles were included if they evaluated malignant skin tumors using LC-OCT and had sufficient information to construct a 2 × 2 table.

### 2.2. Data Selection and Extraction

Titles and abstracts of research studies were assessed by two reviewers. Studies that fulfilled the inclusion criteria were included in the final analysis after full-text screening. From eligible studies, we extracted data for false positive, true positive, false negative, and true negative results of LC-OCT for the detection of malignant skin tumors. Only lesions that were histopathologically confirmed are included in our study. Lesions reanalyzed at the time of follow-up and lesions not confirmed histopathologically were excluded to avoid duplication of lesions. Two studies had one expert dermatologist who reviewed LC-OCT images, one study had two reviewers, one study had four reviewers, and one study did not mention the exact number of reviewers. Studies with multiple reviewers had internal discussions to reach a consensus and to avoid bias. PRISMA guidelines were followed in our study. Our study was registered with PROSPERO (CRD42024494572).

### 2.3. Quality Assessment

To assess the quality and risk of bias of studies, Quality Assessment of Diagnostic Accuracy Studies-2 (QUADAS-2) was used. Four domains covering patient selection, reference standard, index test, the flow of patients through the study, and timing of the index tests and reference standard were assessed via QUADAS-2. The risk of bias was scored as unclear, high, or low.

### 2.4. Statistical Analysis

The sensitivity and specificity of each study were calculated. The pooled sensitivity and specificity with their 95% confidence intervals were calculated using the random-effect model, respectively. The forest plots were constructed. The bivariate summary sensitivity and specificity were calculated using the linear mixed model. The summary receiver operative curve (SROC) was drawn, and the area under the curve (AUC) was calculated. The I^2^ statistic was used to measure the between-study heterogeneity. Statistical analyses were conducted using the mada package of the R language (version 3.5.0 and the web application Meta-DiSc 2.0 [9,10,11].

## 3. Results

### 3.1. Description of Studies Included

**Figure 2:**
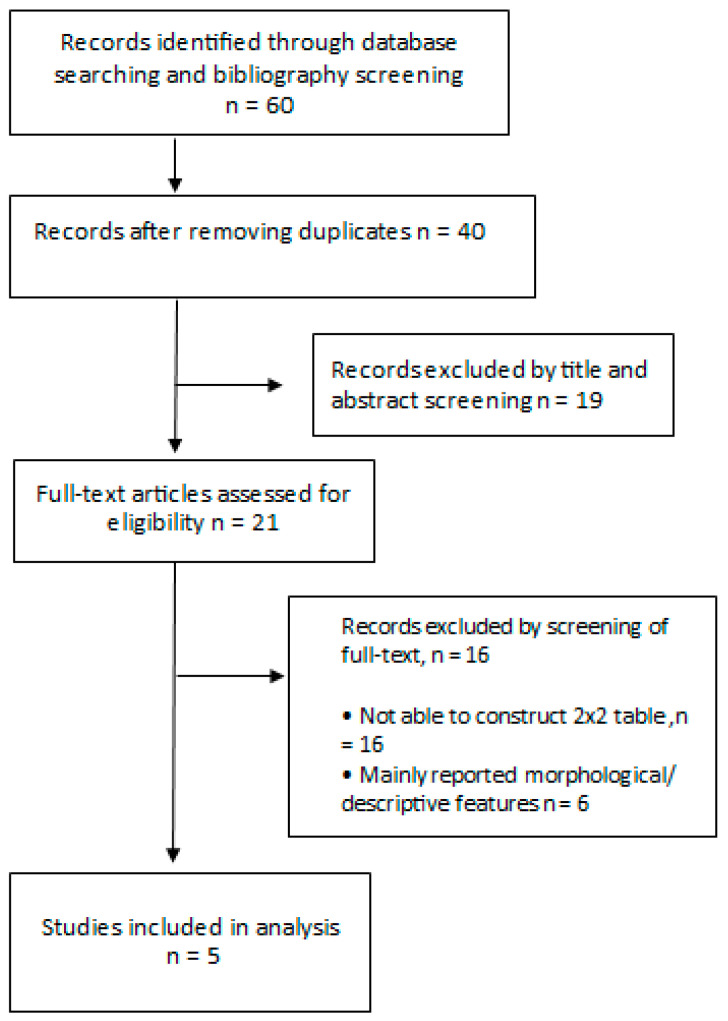
Flow diagram of study selection.

Five studies with a total of 904 lesions were analyzed in this meta-analysis. Relevant characteristics of five studies are described in Table 1 and the selective process is depicted in Figure 2. All studies were conducted in Europe and two of the studies were multicentric. Studies included in this meta-analysis covered most types of skin cancers, including BCC, SCC, melanoma, and AK.

### 3.2. Quality Assessment

Studies included in our meta-analysis were of high quality. We used QUADS-2 to assess the quality of studies. Only one study had an unclear risk of bias in patient selection as it did not specify if patient selection was consecutive or random (Table 2).

### 3.3. Per-Lesion Analysis

**Figure 3:**
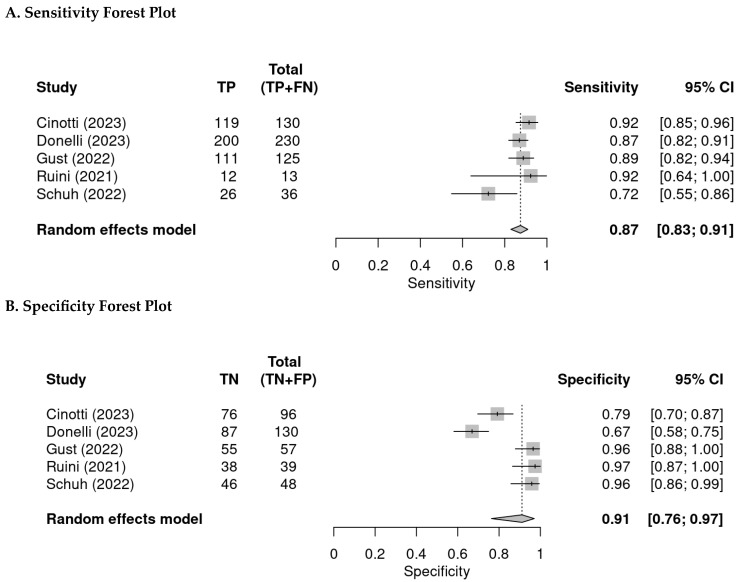
(**A**) Forest plot of pooled sensitivity. (**B**) Forest plot of pooled specificity (TP = true positive, FP = false positive, FN = false negative, TN = true negative) [2,12,13,14,15].

A total of five studies with 904 lesions reported per lesion analyses; the specificity and sensitivity ranged from 67% to 97% and 72% to 92%, respectively. The pooled specificity and sensitivity were 91% (95% CI: 76–97%) and 87% (95% CI: 83–91%), respectively (Figure 3).

**Figure 4:**
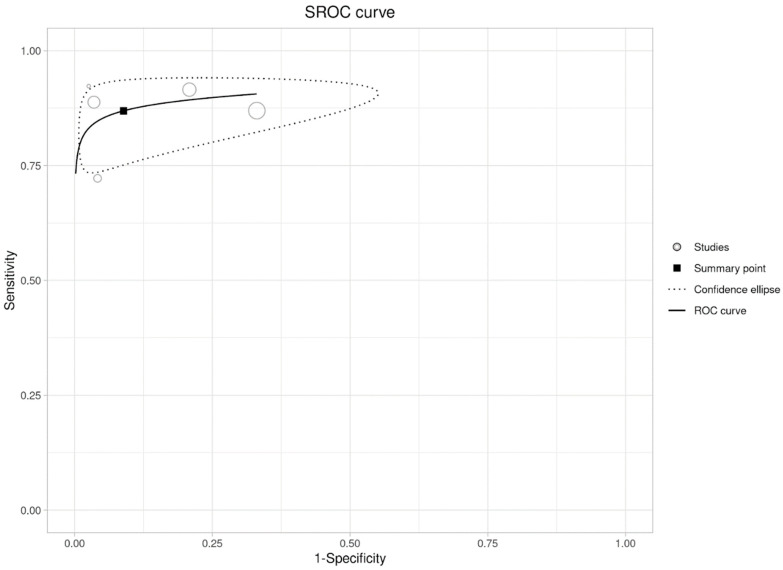
Summary Receiver Operating Characteristic (SROC) Curve for Evaluating the Sensitivity and Specificity.

The summary sensitivity and specificity from the bivariate approach are 86.9% (95% CI: 81.8–90.8%) and 91.1% (95% CI: 76.7–97.0%), respectively. The area under the curve is 0.914. The I^2^ is 38% (Figure 4).

## 4. Discussion

This study is among the first systematic reviews and meta-analyses evaluating the clinical diagnostic efficacy of LC-OCT for malignant skin tumors. Original individual studies lack adequate sample size across limited skin tumors to comment on overall diagnostic efficacy; however, all five selected studies have a low risk of bias and applicability concerns. We find that LC-OCT has shown promise as a clinical diagnostic aid with high sensitivity (pooled 87%) and specificity (pooled 91%) across 904 samples of various skin tumors, including BCC, SCC, melanomas, and premalignant AKs. The larger aggregate meta-analysis allows for a broader analysis of the diagnostic accuracy of LC-OCT for more robust data in support of the clinical utility of LC-OCT.

There are currently numerous diagnostic tools used by dermatologists to identify and diagnose malignant skin lesions. The use of dermoscopy has become standard practice as a screening tool for skin cancer. It has been shown to improve the diagnosis of melanotic lesions and reduce unnecessary biopsies [16,17,18]. The pooled sensitivity and specificity of dermoscopy for the diagnosis of BCC were found to be 91.2% and 95%, respectively, which is slightly improved from our findings for LC-OCT [19]. Errors can occur, however, due to improper use of the dermatoscope, lack of training among practitioners, or incomplete selection of lesions to examine [20]. Histopathology following biopsy is currently considered the gold standard for definitive diagnosis of malignant skin lesions; however, interpretation of histologic samples may vary between pathologists, demonstrating a need for greater standardization [21]. Reflectance confocal microscopy (RCM) has been increasingly studied as a non-invasive imaging modality. It uses a low-level laser for high-power magnification and may be more effective than dermoscopy for diagnosing melanoma [22]. The pooled sensitivity and specificity of RCM were 93.6% and 82.7%, respectively, indicating greater sensitivity but lesser specificity than our findings for LC-OCT [23]. RCM requires formal training in image interpretation, may be expensive to implement, and can be more time-consuming for clinicians than dermoscopy [24].

Another non-invasive imaging technique is conventional OCT which allows for in vivo visualization of skin at a depth up to 1.6 mm. It has shown promise for the identification of BCCs and early neoplastic skin lesions, though its efficacy has not yet been fully established [25,26]. The pooled sensitivity and specificity of OCT for skin cancer diagnoses were 91.8% and 86.7%, respectively, demonstrating, similarly to RCM, greater sensitivity but lesser specificity than our findings for LC-OCT [27]. High-frequency ultrasound has also been explored as a non-invasive visualization modality as it is cost-effective and time-efficient; however, it is unable to provide high-resolution images, and therefore, its use for skin cancers is limited [28,29]. Raman spectroscopy utilizes the Raman spectra of biomolecules as a non-invasive technique for the detection of skin cancers. Its use is limited due to high cost, long image acquisition time, and weak imaging signals [30]. Electrical impedance spectroscopy (EIS) uses the electric properties of skin to determine whether a biopsy is warranted by generating a score from 1 to 10 for skin (scores >3 considered malignant). EIS has, however, been associated with false-negative and false-positive results [31,32].

Infrared thermography is also being studied as a non-invasive method for detecting skin cancer by measuring emitted radiation from the skin’s surface, but further research is required to establish its efficacy [33]. In a pigmented lesion assay (PLA), patches are applied to skin lesions, and genetic material is extracted from the patches for gene assay to detect melanoma. However, PLA cannot be utilized in certain areas of the body and may not work if sufficient RNA is not collected through the patch [34].

LC-OCT aims to combine the advantages of conventional OCT and RCM. LC-OCT can generate vertical, horizontal, and 3D images of similar resolution to the 2D images generated by RCM [1]. Additionally, LC-OCT has a similar vertical view as conventional OCT, with both methods allowing visualization of single cells. LC-OCT has a lower penetration depth than conventional OCT but has a higher resolution [15]. The penetration depth is, however, higher than that of RCM, which is a notable advantage [13].

The use of artificial intelligence (AI) for LC-OCT image analysis is currently being studied. In a study analyzing a deep learning algorithm’s ability to discriminate between healthy and malignant skin, the algorithm achieved an area under the ROC curve (AUC) score > 0.965, compared to an AUC of 0.766 for experienced dermatologists completing the same task [35]. AI has also been successfully used to examine LC-OCT images in a study on skin aging, demonstrating promise for its use in other applications, including skin cancer detection [36].

The diagnostic capabilities of LC-OCT have been explored in other dermatologic therapeutic areas. LC-OCT characterization has been conducted in other neoplasms, inflammatory skin conditions (plaque psoriasis, atopic eczema, and lichen planus), and infectious skin conditions (nodular scabies, herpes, Molluscum contagiosum) [37,38,39,40]. LC-OCT has been utilized to characterize other skin conditions, including pityriasis rosea, scalp psoriasis, Paget’s disease, Hailey–Hailey disease, eccrine poroma, and bullous striae distensae [41,42,43,44,45,46]. As the characterization of many different conditions with larger lesion numbers increases longitudinally, diagnostic accuracy for other conditions may increase. Additionally, the combination of LC-OCT with dermoscopy has improved sensitivity (100%) and specificity (94.9%) in BCC imaging studies, suggesting that multimodal imaging may further improve malignancy detection and characterization.

Biopsy is the gold standard for diagnosing malignant skin tumors. A literature search did not identify a study that calculated pooled sensitivity and specificity for malignant skin tumors, but studies with subgroup analysis were identified. Other competing techniques, such as dermoscopy, RCM, and OCT with pooled sensitivity and specificity analyses for malignant skin tumors were identified and are discussed in detail above. In a study of BCC with punch biopsy, sensitivity was 99% and specificity was 100% [47]. In a study of melanoma, although sensitivity and specificity were not calculated, diagnostic accuracy for invasive melanoma was 95% for excisional biopsy, 77% for punch, and 82% for deep shave [48]. In a study of SCC, although sensitivity and specificity were not calculated, diagnostic accuracy was 83.7% between punch biopsy and surgical excision for TNM staging [49].

The advantages of LC-OCT include being a non-invasive high-resolution 3D imaging technique providing visualization of the epidermis and upper dermis. Real-time, in-office imaging can allow for a more rapid correlation between clinical diagnosis and histopathological correlations, as well as offering in-depth in vivo evaluation of treatment monitoring [1]. Malignant skin tumors currently require biopsy for disease characterization and clinical diagnosis; however, LC-OCT’s diagnostic accuracy may reduce the biopsy burden long term. Additionally, for malignancies treated topically and by field measures (cryotherapy), LC-OCT may gauge treatment efficacy and guide treatment duration [50]. Residual tumors in the deeper layers of the epidermis that may not be visualized by strictly clinical observation may lead to premature discontinuation of therapy; however, LC-OCT may detect residual tumors and guide appropriate treatment.

Non-invasive optical imaging can be used to rule out benign cases, which will prevent unnecessary skin biopsies [51]. Only equivocal cases were sent for biopsy. In our study, the false negative rate is 7.3% (66 out of 904) for malignant skin tumors, but given the limited number of studies included in our meta-analysis, additional larger-scale studies are required in the future. In order to compare the false negative rate between LC-OCT and biopsy results, subgroup analysis of malignant skin cancers should be performed in the future, as this would better highlight the false-negative rate among different subtypes of cancers. In a study with RCM, the false-negative rate was 5% in one study and 3% in another study for melanoma [52].

Potential challenges of LC-OCT include the limited studies evaluating its diagnostic potential in a wide range of malignant skin tumor types. The limited availability and high cost of LC-OCT machines and training associated with image interpretation are limiting factors for its widespread clinical use. Limited field of view and lower penetration depths compared to OCT mean deeper layers of tumor growth may be missed [15]. Areas for future study include larger-scale studies in various malignant skin tumor types and histologies in diverse patient skin types. Integration of AI in LC-OCT imaging for cancer detection may also aid in future image interpretation non-invasively.

The cost of LC-OCT is a prohibitive factor that limits its use compared to other optical imaging technologies like RCM and OCT [1]. In a single-center study with RCM, another optical imaging technique, it was able to avoid 50.2% of biopsies by identifying benign lesions [53]. Similarly, in another study at University Hospital in Europe, 4320 unnecessary excisions were avoided in a year, saving over EUR 280,000 through the use of optical imaging like RCM [54]. LC-OCT can provide similar savings, but data are not yet available given the novelty of LC-OCT. Although LC-OCT is associated with a higher cost of acquiring the device and training medical staff, this will pay dividends in the future as LC-OCT would lead to a reduced number of biopsies over time.

## 5. Conclusions

Based on our study, LC-OCT shows great sensitivity and specificity in diagnosing malignant skin tumors. However, due to the limited number of studies included in our meta-analysis, it is premature to elucidate the true potential of LC-OCT in clinical practice. Therefore, more research is needed to uncover the true potential of LC-OCT. LC-OCT shows great promise as a non-invasive modality to diagnose malignant skin tumors, potentially reducing the biopsy burden. LC-OCT’s clinical utility as a treatment monitoring tool and diagnostic aid may allow for a timely and non-invasive skin evaluation tool.

## Figures and Tables

**Table 1:** Relevant characteristics of selected studies.

Author	Year (Published)	Country	Centre	Type of Cancer	Patients (*n*)	Age (Mean or Median)	Males	Females	Lesions
Cinotti et al. [12]	2023	Italy	One	BCC, SCC, melanomas	196	64.45	115	81	226
Donelli et al. [2]	2023	Italy	One	BCC, SCC, melanomas, AK	NA	NA	NA	NA	360
Gust et al. [13]	2022	Germany	Two	BCC, SCC, AK	154	70.8	102	52	182
Ruini et al. [14]	2021	Germany	One	BCC	52	71	35	17	52
Schuh et al. [15]	2022	Germany	Two	Melanomas	75	51	NA	NA	84

Legend: BCC = basal cell carcinoma, SCC = squamous cell carcinoma, AK = actinic keratosis, NA = Not available.

**Table 2:** QUADAS-2 Risk of bias assessment.

		Risk of Bias				Applicability Concerns		
Study	Year	Patient Selection	Index Test	Reference Standard	Flow and Timing	Patient Selection	Index Test	Reference Standard
Cinotti et al. [12]	2023	Low	Low	Low	Low	Low	Low	Low
Donelli et al. [2]	2023	Low	Low	Low	Low	Low	Low	Low
Gust et al. [13]	2022	Low	Low	Low	Low	Low	Low	Low
Ruini et al. [14]	2021	Unclear	Low	Low	Low	Low	Low	Low
Schuh et al. [15]	2022	Low	Low	Low	Low	Low	Low	Low

## Data Availability

All data generated or analyzed in this study are included in this article. Further inquiries can be directed to the corresponding author.
